# Knowledge-oriented semantics modelling towards uncertainty reasoning

**DOI:** 10.1186/s40064-016-2331-1

**Published:** 2016-06-10

**Authors:** Abdul-Wahid Mohammed, Yang Xu, Ming Liu

**Affiliations:** School of Computer Science and Engineering, University of Electronic Science and Technology of China, Xiyuan Ave, West Hi-Tech Zone, Chengdu, 611731 People’s Republic of China

**Keywords:** Hybrid probabilistic ontology, M2M, Smart home, Uncertainty reasoning, Multi-agent system

## Abstract

Distributed reasoning in M2M leverages the expressive power of ontology to enable semantic interoperability between heterogeneous systems of connected devices. Ontology, however, lacks the built-in, principled support to effectively handle the uncertainty inherent in M2M application domains. Thus, efficient reasoning can be achieved by integrating the inferential reasoning power of probabilistic representations with the first-order expressiveness of ontology. But there remains a gap with current probabilistic ontologies since state-of-the-art provides no compatible representation for simultaneous handling of discrete and continuous quantities in ontology. This requirement is paramount, especially in smart homes, where continuous quantities cannot be avoided, and simply mapping continuous information to discrete states through quantization can cause a great deal of information loss. In this paper, we propose a hybrid probabilistic ontology that can simultaneously handle distributions over discrete and continuous quantities in ontology. We call this new framework *HyProb-Ontology*, and it specifies distributions over properties of classes, which serve as templates for instances of classes to inherit as well as overwrite some aspects. Since there cannot be restriction on the dependency topology of models that HyProb-Ontology can induce across different domains, we can achieve a unified *Ground Hybrid Probabilistic Model* by *conditional Gaussian fuzzification* of the distributions of the continuous variables in ontology. From the results of our experiments, this unified model can achieve exact inference with better performance over classical Bayesian networks.

## Background

*Machine-to-Machine* (*M2M*) (Severi et al. 2014) is an important research area in which smart homes (Zhang et al. 2011; Harper 2006; Jiang et al. 2004; Teymourzadeh et al. 2013) are one of the key areas envisaged to promote its growth. As intelligence in the home takes new shape, multi-agent based context-awareness in M2M (Maracic et al. 2014) performs automated reasoning, which supports interoperability among heterogeneous systems of connected devices using the expressive power of ontology (McGuinness et al. 2004; Staab and Studer 2013; Cardoso and Pinto 2015). An ontology in its classical form, however, is based on *first-order logic*, and lacks the built-in, principled support, to efficiently represent and reason under uncertainty. But we cannot avoid uncertainty in nature, and the fact that knowledge in relation to the entities of the domain can be ambiguous, incomplete, and inconclusive (Orlowska 1998; Yang and Calmet 2005), means that, only a fraction of the rules we use for reasoning can be true in all situations. Particularly, we cannot be sure when to stop amending the set of reasoning rules since not all possibilities can be listed in advance. In probabilistic representation, however, probability can capture uncertain knowledge by summarizing a potentially infinite set of possibilities. Therefore, to achieve compact and efficient reasoning, we can integrate the inferential power of probabilistic representations with the expressive power of ontology to form probabilistic ontology. An ideal form of a probabilistic ontology should be able to comprehensively represent and reason under uncertainty in a principled, structured and shareable way (Boury-Brisset 2003).

Probabilistic extensions to ontology have largely assumed a purely discrete domain of discourse (Ongenae et al. 2013; Yang and Calmet 2005; Maurelli et al. 2013). But ontologies of many real-world problems, especially smart homes, contain continuous quantities such as temperature and humidity. One possible way of handling the continuous quantities is to avoid them by mapping continuous quantities to discrete states through discretization (Pool et al. 2005). But ideally, continuous quantities consist of infinite domains, and subjecting same to discretization often results in intractable conditional probability tables (CPTs) with considerable loss of accuracy. Also, it is usually impossible to define a definite boundary between two overlapping discrete states. We are, thus, faced with a combination of vagueness and uncertainty, and indeed different from the axiomatic notion of probability.

In this paper, we extend the state-of-the-art of probabilistic ontology to include simultaneous distributions for discrete and continuous properties in ontology. To achieve a generic representation, this novel approach leverages the advantages of *probabilistic relational models* (*PRMs*) (Friedman et al. 1999; Getoor et al. 2001), and the fuzzy Bayesian approach of (Pan and Liu 2000), and herein called *Hybrid*(*Hy*) *Probabilistic*(*Prob*-) *Ontology* (*HyProb-Ontology*). The semantics of HyProb-Ontology encodes uncertainty over properties of instances, and uncertainty over the properties of relations between instances of ontological classes. In encoding this uncertainty, we provide representations that simultaneously specify distributions for continuous and discrete property domains in ontology. Thus, for each property of a class in ontology, we specify its probabilistic dependence on other properties of the same object as well as the dependence on properties of related objects. This is a class level probabilistic template that instances of the same class share. Similar to any other class in ontology, subclasses of annotated classes can extend parents’ distributions as well as overwrite some aspects.

Essentially, this framework with its hybrid probabilistic information can induce an equivalent *hybrid Bayesian network* (*HBN*) (Lerner 2002) over completions of instances. Properties of classes serve as random variables in this HBN. Since the dependency topology of the HBN can vary across different domains, pursuing an efficient unified modeling semantics is very vital for future reasoning requirements of M2M. In this regard, our semantics requires replacing each continuous variable in the general HBN with a fuzzy discrete variable, and extending a link from this fuzzy variable to the continuous variable. This fuzzy discrete variable achieves a *conditional Gaussian* (*CG*) *fuzzification* of the continuous variable without employing any *fuzzy logic* (Yager and Zadeh 2012) semantics. We call this generic model a *Ground Hybrid Probabilistic Model* (*GHPM*). In its implementation in HyProb-Ontology, *reference slots* in our *ontological relational schema* are used to achieve the fuzzification of continuous quantities. Note that there is no direct mapping between any fuzzy discrete state and the corresponding continuous variable. However, we associate the Gaussian parameter, $(\mu ,\sigma ^2)$, with each discrete state. To support Bayesian inference in this approach, we provide the algorithm *Construct-GHPM* to aid in constructing the equivalent GHPM from HyProb-Ontology.

We validated this approach using an ontology of a smart home case study. The results of our experiments show that the generic model proposed in this paper can achieve exact inference with better performance over classical *Bayesian networks* (*BN*) (Nielsen and Jensen 2009). Also, pursuing approximate inference algorithms in situations of overly complex networks proves efficient with this approach.

Therefore, the major contributions in this paper are: (1) a hybrid probabilistic model extension to ontology; (2) data structure that achieves generic dependency topology in equivalent DAGs of HyProb-Ontology; (3) algorithm to construct equivalent DAGs of HyProb Ontology; and (4) implementation of uncertainty reasoning in future M2M frameworks.

We organise the rest of the paper as follows: in the following section, we give a brief survey on related work before the research fundamental is presented next; *Hyprob-Ontology modelling* section presents a detailed description of the proposed framework, which is followed by an implementation of a probabilistic reasoning on this framework to achieve uncertainty reasoning in future M2M; *Smart home case study based on oneM2M semantics standards* section presents a case study and an extension of the oneM2M functional model for semantics; next section presents experimental tests and discussions of experimental results; finally, we draw conclusions, and propose future research.

## Related work

Dealing with uncertainty in ontology has extensively been studied for some time now. In most of these approaches, ontological engineering tasks such as domain knowledge modelling, ontology reasoning and ontology mapping (Shvaiko and Euzenat 2008) are expressed along the lines of fuzzy logic and axiomatic notion of probability. The basis for this divide in representation for uncertainty in ontology is attributable to the general notion of information as being vague and imprecise in real application domain.

Just like first-order logic was extended to fuzzy logic, corresponding fuzzy ontology description languages of OWL DL have been proposed to address vagueness in knowledge (Straccia 2006; Bobillo and Straccia 2011). These extend the classical representation of ${\mathcal {SHOIN(D)}}$ to represent domain knowledge using fuzzy sets, and provides reasoning capabilities that support degree of entailment and subsumption relationships in an interval [0, 1]. In demonstrating collective decision making with real scene attributes, a fuzzy ontology based approach has also been proposed (Pérez et al. 2013). With the underlying fuzzy ontology of this approach, relations between concepts and logical rules for reasoning are unified towards efficient decision-making under uncertainty that takes into account large sets of alternatives. We see also an application of fuzzy ontology in recommender systems as a shift from the sundry approaches in information retrieval (Porcel et al. 2015). The adoption of a fuzzy ontology here efficiently characterises users profiles in terms of preferences that accurately generate personalised recommendations. However, fuzzy logic lacks the capacity to deal with inherent uncertainty by exploiting computational structure, which is fundamental to modelling large-scale systems. Taking guidance from statistical relational learning therefore leverages ideas from probability theory to address uncertainty in ontology whilst incorporating probabilistic graphical models (Getoor 2007).

Probabilistic extension to ontology essentially conveys both the structure of probabilistic representation and ontology. In Carvalho et al. (2013), multi-entity BN theories have been implemented towards uncertainty reasoning in ontology under the notion of a discrete domain of discourse. To implement probabilistic subsumption problems for the Bayesian ontology language ${\mathcal {BEL}}$ (Ceylan and Penaloza 2014), a new Bayesian ontology language *BORN* has been proposed (Ceylan et al. 2015). This presents the first Bayesian reasoner over Description Logic ontologies based on multiple world semantics. Also, the literature presents the first approach that considers model complexity of probabilistic ontology in large-scale knowledge-based systems (Mohammed et al. 2015). Novelty of this approach lies in the exploitation of Markov boundary (De Morais and Aussem 2010) in ontology to achieve concepts that prove conjunctively useful for probabilistic modelling.

Even though an attempt has been made through discretization to deal with continuous quantities in probabilistic ontology (Pool et al. 2005), all existing approaches provide no compatible representation for simultaneous handling of discrete and continuous quantities in ontology, and simply mapping continuous information to discrete states through quantization can lead to information loss. In our approach therefore, distributions can be simultaneously specified over properties with discrete and continuous domains in ontology. This representation allows for a derivation of a generic equivalent hybrid *directed acyclic graph* (*DAG*), which provides a unified dependency structure across all domains. Essentially, this unified framework is achieved through conditional Gaussian fuzzification of continuous quantities without employing any fuzzy logic semantics, and can achieve exact inference, which is not typical of classical hybrid probabilistic models.

## Fundamental

In this section, we introduce aspects of the problem of uncertainty reasoning in ontology and hybrid probabilistic models in ontology. These form the fundamental concepts of the main framework presented in the next section.

### Uncertainty reasoning in ontology

Ontologies by default support logic-based reasoning. To incorporate the uncertainty inherent in most real application domains, probabilistic annotations can be added to the classical specification of ontological concepts. Thus, we can formulate a probabilistic ontology as a tuple $\langle O,P_{r} \rangle$, where *O* represents an ontology, and $P_{r}$ denotes associated probabilistic annotations. The problem here is how $P_{r}$ can be encoded to convey both the ontological structure, and the structure of probabilistic representation. With such a conceptualization, existing ontologies can get annotated without any structural adjustments.

Probabilistic ontologies based on PRMs associate probabilistic distributions with properties of classes as value restrictions. If a property *P* has domain *Dom*, we can define its range predicate, *Rg*, as *Rg*(*r*, *P*), where *r* is the range literal of *P*. For most real application domains, *r* can represent discrete or continuous value restrictions of a property. To avoid the tendency of information loss associated with discretization of continuous quantities, distributions can be specified simultaneously for both discrete and continuous quantities in ontology. In this regard, the afore-defined probabilistic ontology can then be transformed into a triple $\langle O,P_{rd},P_{rc} \rangle$, where $P_{rd}$ represents probabilistic annotations of properties with discrete value restrictions, and $P_{rc}$ denotes probabilistic annotations of properties with continuous value restrictions. Using this data structure, we can make probabilistic inferences by translating the probabilistic ontology into an equivalent hybrid DAG. One other looming problem at this stage would be a lack of restriction on the dependency topology of this DAG across different domains. For good model performance, the semantics of these annotations should therefore pursue generic DAGs that can achieve exact inference in practice across different domains.

### Ground hybrid probabilistic model

Motivated by the semantics of PRMs and the FBN approach proposed in (Pan and Liu 2000), we propose a *Ground Hybrid Probabilistic Model* (*GHPM*) that a HyProb-Ontology would induce over *completions*${\mathcal {I}}$ of a relational skeleton, where ${\mathcal {I}}$ represents an instance of a relational schema. This network defines a *conditional Gaussian* (*CG*) distribution: given any assignment to the discrete parents of continuous variables, the distribution over the continuous variables is a multivariate Gaussian (Bishop 2006; Russell and Norvig 2005). Properties of instances of classes in the ontology form the random variables in this framework.

**Fig. 1:**
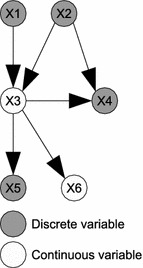
An example of a general HBN. Construction of an equivalent ground hybrid Bayesian network of HyProb-Ontology

Strictly following the semantics of PRMs would require a probabilistic ontology and a given skeleton to induce a classical BN of some discrete ground type. To relax this constraint of a purely discrete domain, HyProb-Ontology specifies distributions over both discrete and continuous variables in the domain. Similarly, given an acyclic dependency topology of a given skeleton, we can construct an equivalent ground HBN of HyProb-Ontology as shown in Fig. 1.

A general HBN is a DAG representing a joint probability distribution over a set of discrete and continuous variables ${\mathbf {X}}$.

1 $$\begin{aligned} HBN=({\mathbf {X,L,P}})=({\mathbf {X}}_{d},{\mathbf {X}}_{c},{\mathbf {L,P}}) \end{aligned}$$

where ${\mathbf {X}}_{d}\in {\mathbf {X}}$ denotes a set of discrete variables, ${\mathbf {X}}_{c}\in {\mathbf {X}}$ a set of continuous variables, ${\mathbf {L}}$ a set of directed edges between variables, and ${\mathbf {P}}$ a set of conditional probability distributions (CPDs). Assuming the set of classes and the relations between them to be fixed, we consider CPD for only properties of classes.

2 $$\begin{aligned} {\mathbf {P}}=\lbrace P(X|P_{d},P_{c}) \rbrace \end{aligned}$$

where $P_{d}$ and $P_{c}$ denote set of discrete and continuous parents of *X* respectively.

Since *X* can be discrete or continuous, obviously, a discrete or continuous variable can have discrete and/or continuous variables as children or parents according to Fig. 1. As a consequence, different topologies with arbitrary probability distributions are eminent. Chances are also that different inference algorithms may be required across different domains, and typical of HBNs, exact inference may not be tractable in most cases.

**Fig. 2:**
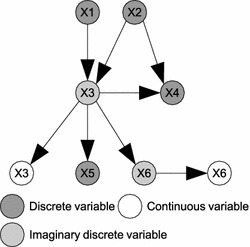
An example of a GHPM from HyProb-Ontology. A generic representation of Fig. 1 that defines hybrid Bayesian networks with fixed topology across domains

As shown in Fig. 2, we present GHPM as a generic representation to check structural arbitrariness in equivalent HBNs of hybrid probabilistic ontologies.

#### **Definition 1**

(*Ground Hybrid Probabilistic Model (GHPM)*) Given the HBN in Eq. 1, GHPM is a conditional Gaussian-class DAG defined by replacing each continuous variable in the model with an imaginary discrete variable, and extending a directed link from these imaginary variables to the corresponding continuous variables.

Based on Definition 1, we can reformulate the general HBN to achieve GHPM as

3 $$\begin{aligned} GHPM=({\mathbf {X}}_{d},{\mathbf {X}}_{dc},{\mathbf {X}}_{c},{\mathbf {L}}',{\mathbf {P}}') \end{aligned}$$

where ${\mathbf {X}}_{dc}$ is the set of imaginary discrete variables obtained after discretization of ${\mathbf {X}}_{c}$ such that $X_{dc}\in {\mathbf {X}}_{dc}\leftrightarrow X_{c}\in {\mathbf {X}}_{c}$, and ${\mathbf {L'}}$ is the new set of links after this transformation. Thus, $\forall X_{c}\in {\mathbf {X}}_{c}$, we replace $X_{c}$ with $X_{dc}$, and a new directed link $X_{dc}\rightarrow X_{c}$ is created. For sake of clarity, we use ${\mathbf {P}}'={\mathbf {P}}_{d}\cup {\mathbf {P}}_{dc}\cup {\mathbf {P}}_{c}$ to represent a composite CPD of the original discrete, imaginary discrete and continuous variables in the transformed HBN.

4 $$\begin{aligned} \left. \begin{aligned}&{\mathbf {P}}_{d}=\lbrace P(X_{d}|P_{d})\rbrace \\&{\mathbf {P}}_{dc}=\lbrace P(X_{dc}|P_{dc})\rbrace \\&{\mathbf {P}}_{c}=\lbrace P(X_{c}|X_{dc})\rbrace \end{aligned} \right\} \qquad \mathbf {\text {P}}' \end{aligned}$$

**Fig. 3:**
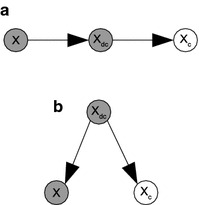
Forms of conditional independences involving continuous nodes in GHPM. **a** Causal chain. This shows that the probability of a continuous variable in the ground hybrid probabilistic model only depends on its imaginary discrete variable. **b** Common ancestor. This implies that discrete and continuous variables in the ground hybrid probabilistic model are conditionally independent given an imaginary discrete variable as their common ancestor

It is worth noting that $X_{dc}$ and $X_{c}$ in the GHPM will always form one of the conditional independencies shown in Fig. 3 with a third discrete node. *Causal chains* shown in Fig. 3a give rise to the conditional independence: $P(X_{c}|X,X_{dc})=P(X_{c}|X_{dc})$. This means that the probability of the continuous variable now depends only on its imaginary discrete variable. Also shown in Fig. 3b is the imaginary discrete variable being a common ancestor to the continuous node and another discrete node. The conditional independence structure of the common ancestor is the same as the causal chains: $P(X_{c}|X,X_{dc})=P(X_{c}|X_{dc})$. This also implies that the two descendants are conditionally independent given their common ancestor. If no information is therefore provided about either descendant, then observing one descendant increases the belief of the ancestor, which in turn increases the belief of the other descendant.

Obviously with this transformation, $P_{d}$ and $P_{dc}$ will have the same configuration comprising of the original and imaginary discrete variables. Thus, their CPDs can be assumed to be multinomial just like in a purely discrete setting. But in a situation of Fig. 3b whereby only $X_{c}$ is observed, the marginal distributions of states of *X* whose beliefs increase with the evidence approximately represents a marginal *cumulative distribution function (CDF)* of *X* given by

5 $$\begin{aligned} F_{X}(x)=\sum _{i\le x}f(i) \end{aligned}$$

where *f*(*i*) represents the discrete probability density function of *X*. The following *lemma* proves this.

#### **Lemma 1**

*Given a discrete variableX*, *and its sibling continuous variable*$X_{c}$*of a common unobserved discrete parent*$X_{dc}$, *the marginal cumulative distribution function ofX*, *given by*$F(X=x)$, *represents increase in beliefs of states ofXwhenever*$X_{c}$*is observed.*

#### Proof 1

Let $F_{X}(x)$ represent the marginal CDF of node *X*, and $F_{X_{c}}(c)$ the marginal CDF of node $X_{c}$. If $\mu$ and $\sigma$ are the mean and standard deviation of $X_{c}$ corresponding to a state $x_{1}$ in the node $X_{dc}$, then increase in belief of $x_{1}$ exists in the interval $[-2\sigma +\mu ,2\sigma +\mu ]$. In effect, the corresponding increase in beliefs in *X* exists in this same interval. Therefore, for monotonic increase in beliefs of states of *X* whenever $X_{c}$ is observed, we can represent the CPD by a conditional CDF. Thus,

6 $$\begin{aligned} F_{X,X_{c}}(X|X_{c})& = {} P(X\le x|X_{c}\le c)\nonumber \\& = {} \frac{F_{X,X_{c}}(x,c)}{F_{X_{c}}(c)} \end{aligned}$$

Given $X_{dc}$, $X_{c}$ and *X* are conditionally independent, and $F_{X,X_{c}}(x,c)=F_{X}(x)F_{X_{c}}(c)$. Hence,

7 $$\begin{aligned} F_{X,X_{c}}(X|X_{c})& = {} F_{X}(x)\nonumber \\& = {} \sum _{i\le x}f(i) \end{aligned}$$

$$\square$$

Each continuous variable, however, now has only one discrete parent $X_{dc}$. With this topology, given any assignment to the discrete variables $X_{dc}$, we can approximate the CPD of each continuous variable $X_{c}$ as a CG given by Eq. 8.

8 $$\begin{aligned} P(X_{c}=c|X_{dc}=d)=\dfrac{1}{\sqrt{2\pi \sigma _{d}}} \exp \left\{ -\dfrac{(c-\mu _{d})^{2}}{2\sigma ^2_{d}} \right\} \end{aligned}$$

where *c* is the value of the continuous variable $X_{c}$, *d* is the discrete state of $X_{dc}$, and the parameter vector $(\mu _{d},\sigma ^2_{d})$ represents the mean and variance of each discrete state *d*. This parameter in essence means there is no one–to-one mapping between *c* and *d*.

9 $$\begin{aligned} P(c|P_{d},P_{c})=\sum _{d} \dfrac{P(d|P_{d})}{\sqrt{2\pi \sigma _{d}}} \exp \left\{ -\dfrac{(c-\mu _{d})^{2}}{2\sigma ^2_{d}} \right\} \end{aligned}$$

Since in the general HBN, Eq. 8 is equivalent to approximating $P(X_{c}|P_{d},P_{c})$ by a conditional Gaussian mixture(CGM) model given by Eq. 9, we can represent the marginal probability, *p*(*c*), of the continuous variable $X_{c}$ by a Gaussian mixture model.

10 $$\begin{aligned} p(c)=\sum _{d} \dfrac{P(c)}{\sqrt{2\pi \sigma _{d}}} \exp \left\{ -\dfrac{(c-\mu _{d})^{2}}{2\sigma ^2_{d}} \right\} \end{aligned}$$

Thus, GHPM can provide a good approximation for the general HBN in ontology since Gaussian mixture models are well known for approximating arbitrary probability distributions (McLachlan and Peel 2004).

## HyProb-Ontology modelling

In this section, we provide details of our hybrid probabilistic model extension to ontology using a smart home semantic control ontology of our case study. The smart home ontology basically models rooms and their properties. This ontology also models devices such as sensors and home appliances, as well as their properties. In line with the essence of our case study, the main properties of devices considered in this ontology are deployment properties, capability properties, and measurement properties.

Following the semantics of PRMs and *Probabilistic Frame-based System*s (Koller and Pfeffer 1998), we begin by providing a relational schema. Similarly, our relational schema represents a direct mapping between ontologies and relational databases. Principally, each class corresponds to a *table*, and properties of a class map to standard attributes in a table. Also, *reference slots*, which correspond to foreign keys in a database are object properties in this framework. Secondly, we give a probabilistic framework to encode dependencies among properties of classes in ontology. This aspect deals separately with the notion of uncertainty in ontology, and how we can represent such uncertainty in ontology.

### Ontological relational schema

**Fig. 4:**
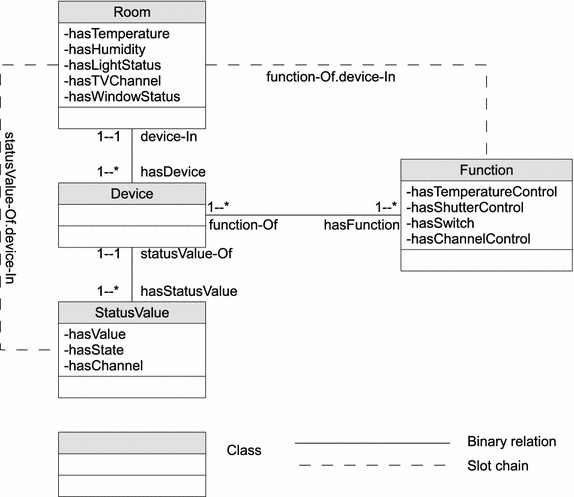
An extract of a relational schema based on our smart home ontology. A logical view of an ontology-based model specifying semantics and their relationships as adopted in this approach

An ontological relational schema describes a set of classes ${\mathcal {X}}=\lbrace X_{1},\ldots ,X_{n}\rbrace$. This is a logical view of an ontology-based model specifying semantics and their relationships. Figure 4 shows a section of the relational schema for the smart home domain considered in this paper.

Each class $X\in {\mathcal {X}}$, has a set of *Properties* denoted by ${\mathcal {P}}(X)$. The Property $P\in {\mathcal {P}}$ of a class *X* is denoted by *X*.*P*, and *V*(*X*.*P*) represents the *value space* of this property. This value space enforces a finite domain on each property object. For example, the class ***StatusValue*** has properties: *hasValue*; *hasState*; and *hasChannel*. Thus, the property ***StatusValue****.hasState* can have a value space $\lbrace On,Off \rbrace$.

Also, a set of *reference slots* of a class *X* is denoted by ${\mathcal {R}}(X)$. We denote the reference slot *R* of *X* by *X*.*R*. Typically, $R\in {\mathcal {R}}(X)$ is of a particular type, and specifies a binary relation between instances of two classes. In essence, each *R* in *X* has a *domain typeX*, and the *range type* is $Y\in {\mathcal {X}}$. Clearly, *R* is an object property of *X*, and can be denoted by *X*.*R*(*Y*). For instance, the relation ***Room***.hasDevice(**Device**) defines a binary relation in which the reference slot *hasDevice* has a domain class ***Room***, and the range type is ***Device***. Additionally, we can define an *inverse reference slot*$R^{-1}$ to denote an inverse function of reference slot *R*. Note that there is no one-to-one mapping between *R* and $R^{-1}$ because the range of $R^{-1}$ is the same as the domain of *R*. For example, the inverse reference slot *device-In* of *hasDevice* has the range type ***Room***, which is domain of *hasDevice*, and domain *Device*, which is also range of *hasDevice*.

Implicit relation between two classes is defined using a *slot chain*, which is denoted by $X.\sigma$ for some class *X*. In this notation, $\sigma$ represents a sequential list of reference slots, $R_{1}.R_{2} \ldots R_{n}$, such that any two successive reference slots must obey the relations $X.R_{i}(Y)$ and $Y.R_{i+1}(Z)$, $\forall X,Y,Z\in {\mathcal {X}}$. Basically, this means, in $\sigma$, the range of a preceding reference slot must be the domain of its succeeding reference slot. Thus, properties of objects defined in terms of their relation to other objects can be referenced using the notation $X.\sigma .P$. We can designate this mode of referencing properties as *property chains*. Here, the first element of $\sigma$ has a domain type of class *X*, and *P* is a property associated with the range class of the last element of $\sigma$. For example, to determine a room’s temperature value, we can use the slot chain ***Room****hasDevice.hasStatusValue.****hasValue***. The referenced property, *hasValue*, is an attribute of *StatusValue*, which is the range class of the last item of the slot chain.

Finally, an *instance*${\mathcal {I}}$ of a schema would specify a set of class objects *x*, a value for each property *x*.*P*, and a value for each reference slot *x*.*R*. In a simplified view, a relational skeleton, which is a partial specification of a relational schema, contains only objects and their relations.

### Uncertainty in ontology

For generic representation of uncertainty in ontology, we extend the idea of probabilistic ontology to include simultaneous distributions for discrete and continuous variables. Building on the framework of PRMs, we specify for each property of an object, the probabilistic dependence on other properties of the same object as well as the dependence on properties of related objects. This is a class level dependency model that objects of the same class can instantiate. Thus, subclasses can inherit as well as overwrite distributions of their parent classes.

Similar to any dependency model, each property *X*.*P* has a set of parents, and a local probabilistic model specifying the dependence on these parents. Note that this probabilistic model is either multinomial or multivariate Gaussian for sake of generality. Two types of parents can be defined for *X*.*P*: dependence on another probabilistic property *X*.*Q*; and dependence on properties of related objects $X.\sigma .Q$. In the latter case where the slot chain may not be single-valued, we can use an aggregate notation $\psi$ to achieve compact modeling. Specifically, *X*.*P* will depend on $\psi (X.\sigma .Q)$, and for any $x\in X$, *x*.*P* will depend on $\psi (x.\sigma .Q)$. We denote the domain of this aggregate function by ${\mathcal {V}}(X.\sigma .Q)$.

Since dependency properties are shared by objects of the same class, we can use CPDs of properties to model dependencies between parent properties and their descendants. Thus, the CPD of *X*.*P* given its parents $P_{a}$ is denoted by $P(X.P|P_{a})$. Each $U_{i}\in P_{a}$ has a domain ${\mathcal {V}}(U_{i})$ in some ground type. Therefore, for the domain ${\mathcal {V}}(X.A)$, we can specify the conditional distribution as $P(X.A|{\mathcal {V}}(U_{i}))$. We can construct the dependency topology of properties in HyProb-Ontology using the following rules:

- a dependency is a pair $X.P\leftarrow Y.Q$ where *X* and *Y* are some classes, *P* is a property of *X*, and *Q* a property of *Y*
- if *P* is a property of *X*, and $Y=X$, then *X*.*Q* can be a parent to *X*.*P*
- if *P* is a property of *X*, then $X.\sigma .Q$ is parent to *X*.*P*, if *Q* is a referenced property in *Y*, and *Y* is the range class of the last item in $X.\sigma$

**Fig. 5:**
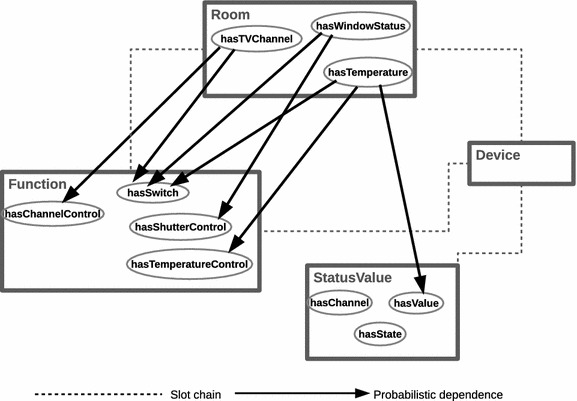
Structural view of probabilistic dependencies between properties in our smart home ontology. Dependency topology of properties in HyProb-Ontology based on the rules provided in this paper

The basic intuition we get from these rules is that this is a class level dependency topology, and all instances consistent with these classes share the same topology. An example of such a dependency topology based on our smart home ontology is shown in Fig. 5. We notice that the reference slots do not have probabilistic dependencies because they are assumed to be known and fixed. Shown as the dash lines, the reference slots only exist implicitly in the topology. It is worth noting also that even though the slot chain ***StatusValue.***statusValue-Of.device-In relates the class *Room* to another class *StatusValue*, only one probabilistic dependence exists between them. The idea is that properties of these two classes are equivalent, and we choose to ignore the inter-discrete dependencies in favour of our approach. This explains the flexibility of HyProb-Ontology to partially define distributions over properties of classes.

### Encoding hybrid probabilistic information in ontology

**Fig. 6:**
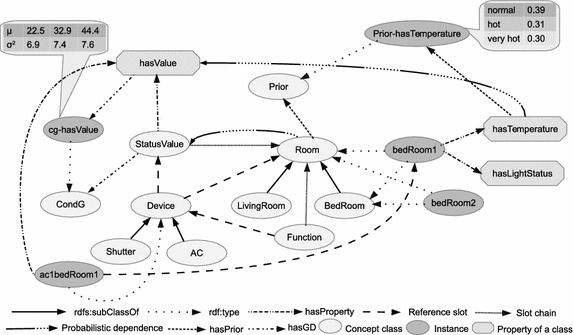
An example of annotation based on HyProb-Ontology. A high-level view of the annotation of hybrid probabilistic information in ontology

The ability to associate distributions with classes and their properties is key towards achieving probabilistic extension to ontology. To augment the standard OWL elements with this uncertainty information, we define probability and reference slot as resources in ontology. An example of an annotated ontology based on HyProb-Ontology is shown in Fig. 6.

Under the HyProb-Ontology framework, a mandatory class: *SlotChain*; is required to represent slot chains in ontology. For simplicity, every binary relation in this representation is considered a slot chain of the form $X.\sigma .P$ such that

$$\begin{aligned} X.\sigma .P = {\left\{ \begin{array}{ll} X.P, &\quad{} \text {if }\sigma =\emptyset \\ X.\sigma .P, &\quad{} \text {if }n(\sigma )>=1 \end{array}\right. } \end{aligned}$$

In this sense, a simple reference slot is also considered a slot chain with cardinality of $\sigma$ being 1. Since $\sigma$ denotes a sequence of reference slots, a property, *element*, is required to list elements of $\sigma$. Each element of $\sigma$ is, thus, described using another property *slot*, and its index in the sequence is maintained by the property *slotIndex*, which is rdf:datatype integer. Note that no referenced property is used in this representation because a class instance holds this relation, and every property *P* of the class shares the same relation. All reference slots in the relational schema are therefore instances of this class. For example, the reference slot *Device.device-In* can be represented in ontology as shown in Listing 1.

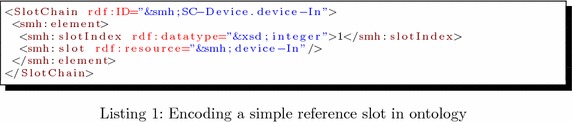



Also, the slot chain *StatusValue.statusValue-Of.devive-In* can be represented in ontology as Listing 2. Obviously, what divides the two representations is the number of element blocks in their respective representations. Hence, to link properties of a class with other properties of relations, we require the definition of a holder of the slot chain resource using the property *hasSlotChain*. This property becomes useful only when inter property dependence is required.

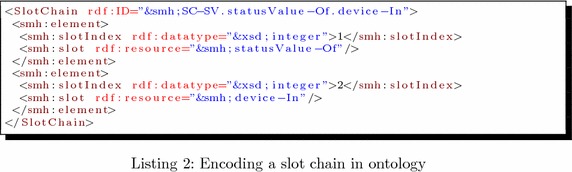



The idea that an object’s property can depend on other properties of either the same object or properties of related objects indicates the possibility of prior and conditional distributions existing within a class. In this case, our semantics must provide means for annotating properties with prior and conditional probability distributions. Therefore, to represent probability in ontology, we define three mandatory classes: *Prior*; *CondP*; and *CondG*; to respectively represent prior probability distribution, CPT, and CG distribution as possible restrictions for probability values of properties of classes. A class containing an annotated property is, thus, termed a probabilistic class, and we will be using *p-Class* to denote same.

To specify the hybrid distribution we seek in this paper, we have to specify three kinds of distributions in ontology: prior distribution for properties with discrete value space; multinomial distribution for properties with discrete value space given discrete parents; and CG distribution for properties with continuous value space given discrete parents. In establishing this generic representation, we define the property, *isDiscrete*, which takes the value *True* or *False* in order to simultaneously handle distributions over discrete and continuous variables in ontology.

#### Discrete distribution

As shown in Listing 3, the prior probability restriction resource of a property is an instance of the Prior class. The target property of this distribution is specified using the attribute *property*. Similar to the representation of a slot chain, probability values of items of a property’s value space are partitioned using the property *element*. We provide also the property *domainElement*, which describes the elements of the annotated property’s value space. We specify values of the elements of this value space using the property *pValue*, which is rdf:datatype double. To ensure that the axioms of probability hold, sum of all values, which are specified by pValue of any instance must equal 1.

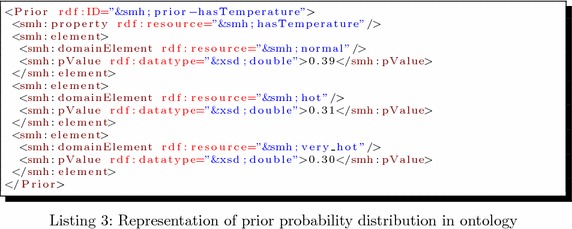



For a property having a discrete value space, the conditional probability distribution is represented using a CPT, which is an instance of the class CondP. An example of HyProb-Ontology’s representation of CPT in ontology is shown in Listing 4. As can be seen in this listing, the structure of the conditional probability distribution is similar to the prior distribution. The only difference between these two representations is that the attribute, domainElement, in the definition of the prior distribution is replaced with another property *rowCell*. Since we seek a tabular representation of the CPT, the rowCell attribute defines the item combinations of the value space of the property over values of its parents. In each element block, the number of resources defined by rowCell is subject to the number of parents a property has. Thus, the number of resources specified by rowCell in each element block is equal to $n+1$, where *n* is the number of discrete parents of a property. In the example of Listing 4 for instance, the property *hasShutterControl* has a single parent, and so each element block accordingly defines two rowCell resources.

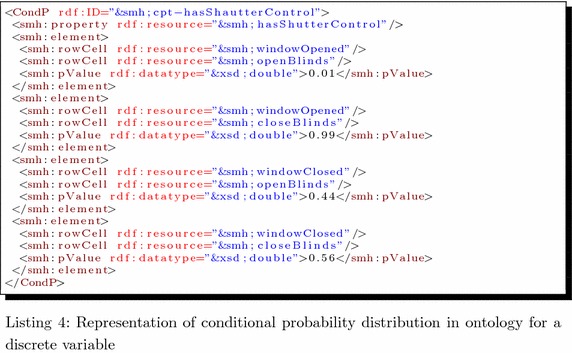



Annotating a given property with a discrete prior distribution requires a property *hasPrior*. The value of this property gives the prior distribution restriction, which is also a resource defined in the ontology. Also required is the property, isDiscrete, whose value is set to True. For instance, the annotation of *Room.hasWindowStatus* can be represented in ontology as shown in Listing 5.

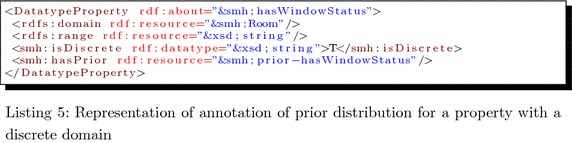



Similarly, a property is annotated with a conditional probability distribution just like the annotation of the prior distribution. In this case, the property, hasPrior, in the annotation of the prior distribution is replaced with the property *hasCPT*. Additionally, we augment the annotation of the prior distribution with the properties *hasParent* and *hasSlotChain*. We show in Listing 6 an example of annotation of conditional distribution based on the semantics of HyProb-Ontology. From this representation, hasParent is required to specify parents of the annotated property. Also, the relations between a property’s class and those of its parents are specified by the property hasSlotChain. As clearly demonstrated in the annotations of both the prior and conditional distributions in ontology, our approach achieves compact representation similar to the standard OWL definition of properties.

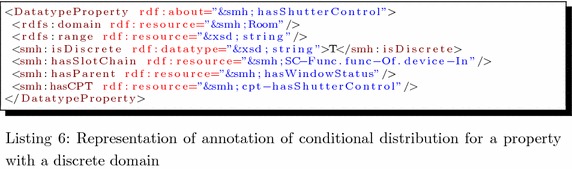



#### Continuous distribution

From Definition 1, the generic framework we are seeking requires an ontological representation that can achieve CG distribution over properties whose value spaces are continuous. In this pursuit, all continuous quantities in the domain should have multivariate Gaussian distributions, i.e. CG, of the type CondG. Thus, for each property with a continuous value space in ontology, we specify two types of distributions: discrete distribution over an *equivalent* property that has a discrete value space of the continuous domain; and a CG distribution over the continuous value space of the property. This fictitious (equivalent) discrete property serves as the sole parent of the continuous property in the equivalent DAG of HyProb-Ontology. The fictitious discrete property hereby replaces the continuous property in all dependencies in the ontology, and its representation follows those of the discrete setting.

Even though we can use *owl:equivalentProperty* to create the fictitious property in ontology, slot chains in HyProb-Ontology do the trick easily. Whilst the continuous property is an attribute of the domain class of the slot chain, the fictitious property exists in the range class of the last item in the slot chain. In essence, CG fuzzification of continuous quantities is achieved in HyProb-Ontology using implicit relations between continuous quantities and their corresponding discrete properties. Thus, for a property chain $X.\sigma .Q$; *Y*.*Q* is considered a fictitious discrete equivalent of *X*.*P* if only the continuous domain of *X*.*P* corresponds to the discrete domain of *Y*.*Q*, and *Y* is the range class of the last reference slot in the chain. As shown in Fig. 5, with a typical oneM2M (Sneps-Sneppe and Namiot 2012) framework for example, slot chains can provide the dependence of the value of a temperature sensor(continuous quantity) on the temperature states of a room(discrete states). As an advantage, the adoption of slot chains in this approach can greatly simplify the process of semantic annotation in the generic oneM2M functional model for M2M applications. For instance, *hasTemperature*, which gives the interpretation of the continuous value of the property, *hasValue*, of a temperature sensor is inherently discrete. Clearly, these are two equivalent properties that represent the same phenomenon differently using discrete and continuous quantities. Therefore, hasTemperature can be the fictitious discrete equivalent, and also the only parent of hasValue in the dependency model.

The conditional distribution of properties with continuous value space is defined as an instance of the class CondG. Similar to the conditional distribution of discrete properties, only the property pValue in the definition of the prior distribution of discrete properties is dropped. We also introduce two additional properties: *mean*; and *std*. Just like any Gaussian distribution, the properties mean and std respectively represent the mean and variance of the distribution. These two properties form a parameter vector that the semantics associates with each discrete state in the domain of a corresponding equivalent fictitious property. We specify each corresponding discrete state of the parameter vector using the property domainElement. Shown in Listing 7 is a representation of CG distribution of the property hasValue in ontology. As you can see, we associate a parameter vector consisting of the mean and variance with each state of the fictitious discrete property. Clearly, this data structure achieves a multivariate Gaussian distribution with mean, $mean\in \mathbf R ^n$ and covariance matrix $std\in \mathbf S ^n$, where *n* is the length of the discrete states of the fictitious discrete property. Therefore, the dimension of the covariance matrix depends on the number of discrete states of the fictitious discrete variable.

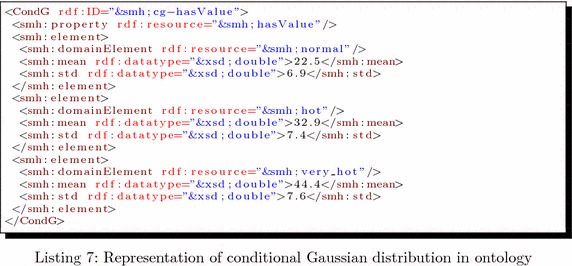



Similar to the annotation of conditional distribution of discrete properties, the conditional distribution restrictions in the annotation of properties with continuous value space are defined using the property *hasGD*. This replaces the property hasCPT in the annotation of discrete conditional distributions, and holds the resource of the Gaussian distribution associated with the property. In this setting of continuous domain also, we are required to set the property value of isDiscrete to False. An example of representation of the annotation of a property with a CG distribution is shown in Listing 8. Note that, unlike in the annotation of conditional distribution of discrete properties, this structure is fixed for all continuous domains of properties. Thus, whereas multiple parents are possible in the annotation of conditional distributions of discrete domains, the annotation of continuous properties always has the fictitious discrete property as the sole parent.

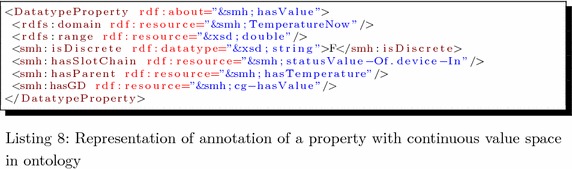



Just like any other class in ontology, the inheritance mechanism provides for one annotated class to be a subclass of another. This allows subclasses to extend specifications of parent classes as well as overwrite some aspects of them. Specifically, a subclass can redefine the probability model of one or more properties in the parent template. For example, the class *Bedroom*, which is a subclass of *Room* can inherit the template probabilistic model of a room’s property, *hasTemperature*. Different bedrooms, however, can modify this parent distribution differently. Also, since an instance of a subclass is also an instance of the superclass, it can, thus, fill a property whose type is the superclass. This allows instances of subclasses to be referenced directly using properties of the superclass.

## Probabilistic reasoning on HyProb-Ontology

In this section, we show how probabilistic reasoning based on HyProb-Ontology can achieve uncertainty reasoning in our extended generic functional model for supporting semantics in M2M applications. This can be represented by a piece of software that can support both probabilistic rule-based reasoning and Bayesian reasoning. Whilst the Bayesian reasoner propagates beliefs based on property values, the rule-based reasoner can infer new implicit knowledge not explicitly expressed in ontology.

### Rule-based reasoning

The rule-based reasoner consists of a probabilistic rule base, which can be interpreted by merging probabilistic rules with semantically annotated data and probabilistic knowledge base. In context, a probabilistic rule is an ordered pair of properties of ontological concepts, which has a left-hand side and a right-hand side, and annotated with probabilistic information. Note that this rule base has a predetermined, total ordering to provide support for sequential execution of rules. Since the rule base is open to inclusion of new rules, reordering must always be performed to maintain the right order of execution whenever an update on rules occurs.

**Fig. 7:**
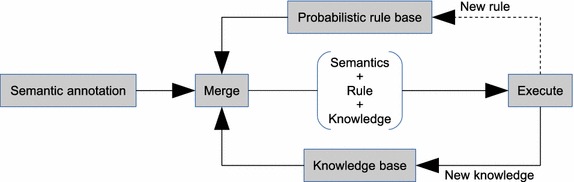
Logic flow of the rule-based reasoner. Illustration of the basic operation of a rule-based reasoner

Figure 7 illustrates the basic operation of the rule-based reasoner. In interpreting rules, the reasoner first performs ontological matching by scanning the left-hand side of each rule using the semantically annotated data until a successful match is found in the knowledge base. This is followed by beliefs updating in which property values of matched instances of concepts are set based on evidence from the semantically annotated data. At this point, the right-hand side of the rule is updated with the property’s value with the highest probability, and the process either continues with the next rule or begins again with the first rule. To infer new implicit knowledge for example, a simple rule could be that the light status of a room can establish states of the room’s bulbs. If for instance, we observe the room to be bright, then the inference would be based on: $Pr(hasLightStatus(Room,Bright),1.0)\Rightarrow Pr(hasStatusValue.hasState(Bullb,On),0.8)$. This rule in probabilistic representation is the same as

$$\begin{aligned} P(hasState|Room,hasLightStatus=Bright). \end{aligned}$$

The advantage with this approach is that this same rule requires no modification in order to reason about the antecedent given the consequent as evidence. Thus, a probabilistic rule can be viewed as a conditional probability statement, and the Baye’s theorem offers such flexibility for manipulations. Hence, the invocation of probabilistic rules can be described as a sequence of actions chained by *modus ponens* (Buchanan et al. 1984).

Similarly, we can reason about concepts not explicitly expressed in ontology using this same reasoner. In this case, implication propagation based on predefined rules over instances is the working principle of the reasoner. For example, with the reference slot $ac1bedRoom1.device-In.Y$, we can infer from the ontology that *Y* must be of type Room to ensure satisfiability.

### Bayesian reasoning

Bayesian reasoning can be used to answer queries about values of properties based on the GHPM. To facilitate automatic discovery of GHPM from HyProb-Ontology, we propose an algorithm *Construct-GHPM*. In this algorithm, we denote the DAG by *G*, and each node in G has the form *I*.*P*, where *I* represents an instance of a p-Class and *P* is a property of a class. The algorithm maintains the list *L* of all nodes to be processed. To begin, *L* is initialized to only properties of named instances. In each iteration, the algorithm removes a node from *L*, and processes it as follows: for each parent $I.\sigma .P_{i}$, an edge is added from $I.\sigma .P_{i}$ to *I*.*P*; if $I.\sigma .P_{i}\notin G$, add $I.\sigma .P_{i}$ to *G* and *L*; when parents of *I*.*P* no longer exist, and depending on the value of isDiscrete in the annotation of *I*.*P*, CPT or CG is then constructed using the hasCPT or hasGD restriction of *I*.*P*.

Now with the network constructed, the Bayesian reasoner can infer probabilities of nodes given evidence. Update of beliefs in the network propagates belief updating of properties in the probabilistic ontology.

#### Inference

Probabilistic inference can be done on an acyclic and a fixed structure GHPM. The process here is similar to first-order logical inference using propositional inference on equivalent propositional knowledge base. Even though our semantics guarantees exact inference with the GHPM, it makes sense also considering approximate inference methods because in large and densely connected networks, the exact inference becomes intractable. Approximation algorithms such as *Markov Chain Monte Carlo* (*MCMC*) (Russell and Norvig 2005), are therefore useful for inference in HyProb-Ontology. These algorithms provide approximate answers whose accuracy grows with the number of samples generated.

To make inference on an acyclic and a fixed structure GHPM using MCMC, the algorithm samples from the possible worlds defined by value spaces of properties of instances in HyProb-Ontology. MCMC generates each event by randomly changing the preceding event. Thus, it performs random movements around the state space, flipping one variable at a time whilst maintaining the evidence variables.

## Smart home case study based on oneM2M semantics standards

As M2M becomes more pervasive (Wu et al. 2011; Zhang et al. 2011), oneM2M is the current vendors’ standards association that envisions a standardized connected devices platform. Semantics in oneM2M are required to provide machine interpretable descriptions using meta-data and annotations. To demonstrate feasibility of uncertainty reasoning in the oneM2M generic functional model for semantics (oneM2M 2014), we consider in this paper a case study of a smart home based on the oneM2M *Use Case on Semantic Home Automation Control System* proposed in technical report *TR-0007* (Zhang and Ju 2014).

### Extended generic functional model for supporting semantics

As one of the three main functional blocks of the generic oneM2M functional model for semantics, this paper is focused on *Abstraction and Semantics*. Specifically, *Semantic reasoning* and *Ontology modelling* are our main focus. Semantic reasoning, of course, is of interest because it has been identified as one of the key technologies that can support semantics in oneM2M.

**Fig. 8:**
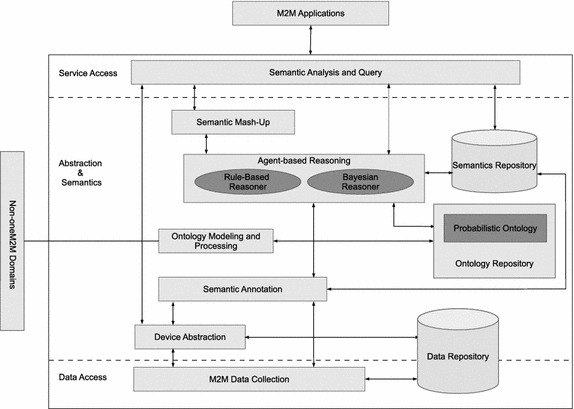
Extended generic functional model for supporting semantics in M2M applications. This shows our version of the oneM2M generic functional model for semantics in an attempt to addressing uncertainty reasoning in future M2M applications

As shown in Fig. 8, we propose an extended oneM2M generic functional model for semantics. In this model, *Common Services Entities* constitute a multi-agent system, whose operational and environmental dynamics are described using semantics. Because uncertainty in nature is unavoidable, and coupled with the lack of complete knowledge about the world, incorporating uncertainty into the semantics can enable these agents’ support for decision-making under uncertainty. As such, it is important that domain uncertainty is considered during the process of ontology modelling since ontological representation form the underlying semantics of this model. In this regard, this new architecture, which is based on our HyProb-Ontology, handles uncertainty during ontological modelling by incorporating probabilistic information. As shown in the architecture, the product of this uncertainty-based ontological modelling is a repository for probabilistic knowledge bases, which are semantically connected to support interoperability. Thus, this repository can guarantee storing, retrieving and maintaining probabilistic knowledge bases, and can merge with semantically annotated data to support uncertainty-based distributed reasoning. New implicit knowledge based on semantically annotated data can therefore be derived taking into account both domain and context uncertainties.

### Case study

We consider a semantic home automation control system as a case study. In this case study, a home’s energy consumption can be minimised by autonomously adjusting the home’s devices to attain optimal operations. Lighting control, temperature control and window control are some of the functionalities of this scenario. Though resource consumption is key in the control strategy of this system, comfort of the home’s inhabitants can not be overlooked too. Therefore, we are confronted with some level of uncertainty over minimizing the home’s energy consumption whilst maintaining the comfort of the home’s inhabitants.

For instance, on a cool sunny day, the smart home automation system might find it optimal to conserve energy by opening windows, turning air conditioners off, and turning lights off. What is uncertain is whether such a strategy would always ensure the comfort of the user. Particularly, opening windows to achieve favourable indoor temperature when the outdoor air is heavily polluted can cause a lot of discomfort to the user. Also, when the environment is noisy, and this strategy is employed whilst the inhabitants are studying would be undesirable. Clearly, uncertainty would always exist in nature, and how best we manage that determines the effectiveness of these technological systems.

## Results and discussion

This section presents description of experiments, and analyses of results to show efficiency of our approach. In our experiments, we constructed two probabilistic ontologies: one based on HyProb-Ontology; and another based on discretization of continuous quantities in ontology(classical probabilistic ontology). It is important to note that both probabilistic ontologies used the same smart home ontology of our case study. And since propagation of beliefs based on property values of concepts handles uncertainty in the probabilistic rule-based reasoner of our extended oneM2M functional model for semantics, we considered only Bayesian reasoning in our experiments. In this regard, we constructed an equivalent GHPM of our HyProb-Ontology, and an equivalent classical BN of the classical probabilistic ontology to perform probabilistic inferences. In these DAGs whereby *n* continuous quantities exist in the ontology, our GHPM would contain 2*n* nodes more than the classical BN. Thus, where as the GHPM is a hybrid probabilistic model, the classical BN contained only discrete nodes. We performed all experiments using an HP Workstation with specification given in Table 1.

**Table 1:** Specification of computer for experiments

Description	Parameter
Processor	Intel(R) Core(TM) i5-3470
	CPU @ 3.20 GHz
RAM	8 GB
System type	64-bit Operating System

To validate the performance of our approach, we performed inferences on our GHPM and the classical BN using marginal distributions of equivalent nodes in both graphs. Discrete states in the classical BN, continuous values in the GHPM, and continuous values together with discrete states of the fictitious(fuzzy) discrete variables in the GHPM were evidences considered in the experiments. To begin with, we generated three different samples:100; 500; and 1000; and learned model parameters of each DAG based on each sample using maximum likelihood parameter estimation. One key condition we imposed on these experiments was to guard against inconsistences in priors of equivalent nodes in the DAGs. In particular, parameters of the CG nodes in the GHPM were learned to be consistent with the range of values specified in the discretization of corresponding discrete nodes in the classical BN. Specifically, the fuzzy discrete node of a continuous temperature node in the GHPM is ancestor to both the continuous node and a discrete node for temperature control. In this case, temperature values of the continuous node, representing the comfort index of a room, were discretized into three states: *normal*; *hot*; and *very hot*; in the equivalent node of the classical BN. Each discrete state spanned a range of continuous values, and directly affected states’ beliefs in the temperature control node during the experiments. For example, we defined a normal temperature state in these experiments to be the range (18–27 ºC), and used a mean of 22.5 in the Gaussian parameters of its continuous node in the GHPM. Thus, all things being equal, using the state normal in the classical BN, or any continuous value in the range of this discrete state as evidence, the influence on states’ beliefs of the temperature controller’s node should be the same.

**Fig. 9:**
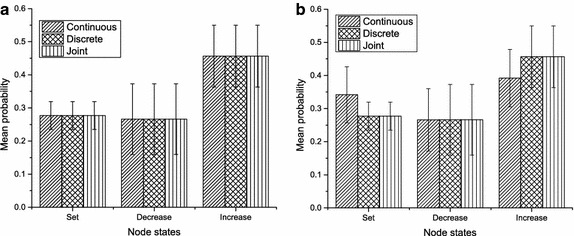
Node distributions. Illustration of marginal distributions of the same node based on our GHPM and classical BN, **a** marginal distribution below mean. Using values below the mean as evidence, both the GHPM and classical BN give approximately the same results. **b** Marginal distribution above mean. Using values above the mean as evidence, results obtained using GHPM significantly differ from the results of the classical BN

In all analyses, the probability values represent average values over all the three samples considered. Figure 9 illustrates the marginal distributions of the same node based on our GHPM, and the classical BN. As expected, given a state of a node as evidence, and keeping other variables put in the classical BN, we obtained constant marginal distributions for states of the target node. In the GHPM however, we partitioned the evidence into values below and above the mean of the Gaussian distribution of the CG node. When we considered values below the mean as evidence, we obtained the results shown in Fig. 9a, which is inconspicuously different from the marginals obtained with the classical BN. This variation in marginal distributions becomes more obvious above the mean value, and gives indication of some loss of information inherent in discretization of continuous variables. Specifically illustrated in Fig. 9b, when values above the mean value, 22.5, were used as evidence in the GHPM, we obtained impressive results in the marginal distributions with the GHPM as compared to both the classical BN, and joint distribution involving both the continuous value and a corresponding fuzzy discrete state in the GHPM. In these DAGs, *set* is the temperature controller’s state that a normal temperature value should directly influence. Given a continuous value as evidence for normal temperature in GHPM, it is obvious from Fig. 9b that GHPM produced the highest increase in belief for this state. Also, the belief of *Decrease* is influenced more by the states: hot; and very hot. By observing this state, we notice that all three distributions approximately overlap, and the edge of GHPM can be seen in our choice of evidence, 27. This evidence is slightly beyond −2 standard deviations of the mean of the state hot (28–38), and thus would have less effect on this state. In the last state too, GHPM produced the least marginal distribution compared to both the classical BN and the joint distribution. When we compare this distribution to the marginal distributions in Fig. 9a, this is a good observation because the range of this state is approximately within $-4$ standard deviations of the evidence, and reduction in belief is realised.

**Fig. 10:**
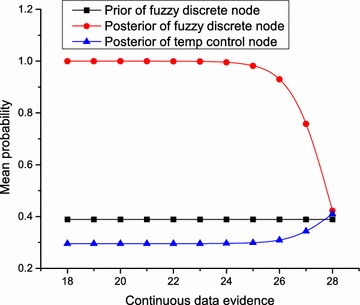
Propagation of belief using continuous data as evidence. This shows that the joint distribution involving the continuous node and its fuzzy discrete node is the same as using the fuzzy discrete state alone as evidence

In the GHPM, joint distribution involving the continuous node and its fuzzy discrete node is the same as using the fuzzy discrete state as evidence. As shown in Fig. 10, when the fuzzy discrete node is unobserved, any continuous value used as evidence increases the belief of the fuzzy discrete node, which in turn increases the belief of the temperature controller node. If we examine these graphs carefully, we will notice that within −2 standard deviations of the mean of our defined normal temperature range, any given continuous value evidence would increase the marginal distribution of the normal temperature state of the fuzzy discrete node. This increment is above the prior probability and even very close to certainty, i.e. approximately 1. Whilst this increases, the marginal distribution of the state of the temperature control node also increases slightly. Interestingly, from the mean value to the point of +2 standard deviations of the mean, whilst the probability of the state of the fuzzy discrete node decreases sharply, the probability of the state of the temperature control node also increases sharply until both approximately converge to the prior probability of the state of the fuzzy discrete node. At this point, the posterior distribution of the fuzzy discrete state is the same as the likelihood when this state is not observed, and the posterior distribution converges to the prior distribution.

If however, the fuzzy discrete node is observable, any additional continuous value used as evidence would have no effect on the distributions of the temperature control node. Hence, the discrete node and the continuous node are conditionally independent given the fuzzy discrete node, and the effect of the joint distribution is the same as that of the fuzzy discrete node only.

**Fig. 11:**
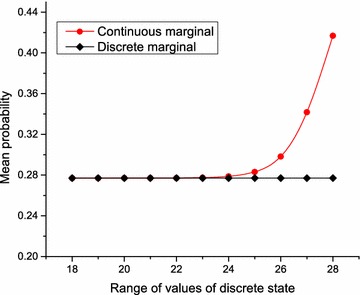
Marginal distributions obtained with GHPM and classical BN. With the same experimental condition, comparisons between results of GHPM and classical BN show the extent of information loss through discretization of continuous variables

To gain more insight into the extent of information loss through discretization of the continuous variable, we measured the marginal distributions of a state of the temperature control node using the range of values in the domain of normal as evidence. As shown in Fig. 11, the distribution of the continuous evidence follows the pattern of a CDF, and the variation becomes more significant between the mean value and +2 standard deviations of the mean value. What we can infer here is that discretization reflects perhaps the true state of the measured quantity within −2 standard deviations of the mean.

**Fig. 12:**
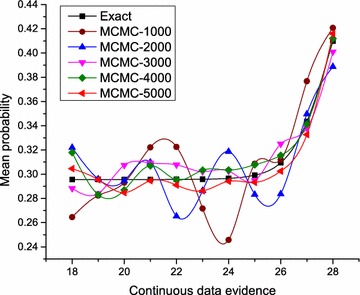
Inference on GHPM using MCMC. This shows the performance of Hyprob-Ontology using approximate inference methods for large scale knowledge bases

We concluded our experiments by examining the performance of MCMC-based inference on GHPM. As shown in Fig. 12, you notice that the accuracy of the MCMC based inference approaches the exact solution as more samples are considered. If we consider the cases of 1000 and 5000 random samples for example, it is clear from this figure that the latter is much closer to the exact solution than the former. This approximate approach, thus, proves useful for HyProb-Ontology when large-scale knowledge bases are encountered.

From above discussions, it is obvious that even though BNs remain powerful in the design of expert systems in uncertainty frameworks, the required discretization of continuous quantities in real systems makes BNs less efficient compared with this paper’s approach. Even if clear boundaries exist between discrete states obtained from continuous nodes, filling the CPT of a node requires up to $O(2^{k})$ combinations. This presents a great deal of intractability, which is overcome in the standardised pattern of our GHPM by supplying much easier fewer parameters than this exponential number in the BN. Since the equivalent DAG of HyProb-Ontology is GHPM, many advantages therefore come with this design.

A key advantage of our approach is to provide a better alternative to the full discretization of continuous data in probabilistic ontologies. Once we seek to solve M2M real domain problems, continuous quantities are unavoidable, and application of continuous models instead of discrete models can avoid information loss due to discretization for better performance in practice. Additionally, the unified dependency model that this framework presents is essential for cross-domain computations in oneM2M. Instead of employing different algorithms to deal with different domains with varying dependency topologies, the generality of this framework allows us to use a single algorithm, which can achieve exact inference across board.

Even though this approach proves efficient empirically, the directed dependency topology of the underlying DAG of HyProb-Ontology is not suitable for modelling cyclic relations in knowledge. Another limitation of this approach is the inability of our semantics to represent relational uncertainty at this stage. Once we relax the fundamental notion of relations being known and fixed a priori, uncertainty in relations between class objects is essential for model accuracy.

## Conclusion

In an attempt to curtail the loss of information inherent in discretization of continuous quantities, we have proposed a hybrid probabilistic model extension to ontology. In this framework, probability distributions can be specified simultaneously over properties with discrete domain, and properties with continuous domain in ontology. This presents a class level probability template that instances can inherit as well as overwrite some aspects. To restrict the dependency topology of the equivalent hybrid DAG that can be constructed from HyProb-Ontology, this approach achieves a generic representation by conditional Gaussian fuzzification of continuous quantities in ontology. Based on the results of our experiments, this approach can achieve exact inference with better performance over classical Bayesian networks. Future work of this research shall consider relational uncertainty currently not implemented in order to relax the constraint that objects and their relations are known and fixed a priori. This assumption is not practical, especially in oneM2M, where the number of connected devices and their relations can not be fixed. Also, since the acyclicity constraints of DAGs limit their applicability to relational data due to the cyclicity problem, future work shall exploit undirected models, which are capable of handling cyclic relations in data.
